# Modeling of Heat Transfer through Firefighters Multilayer Protective Clothing Using the Computational Fluid Dynamics Assisted by X-ray Microtomography and Thermography

**DOI:** 10.3390/ma15155417

**Published:** 2022-08-05

**Authors:** Morgan Renard, Adam K. Puszkarz

**Affiliations:** Lodz University of Technology, Faculty of Material Technologies and Textile Design, Institute of Material Science of Textiles and Polymer Composites, 116 Żeromskiego Street, 90-924 Lodz, Poland

**Keywords:** biophysical comfort, thermal insulation, heat transfer, textiles, protective clothing, CAD modeling, simulation, thermography, micro-CT

## Abstract

This paper explores the modeling of physical phenomena that occur in clothing that affect the safety and biophysical comfort of the user. Three-dimensional models of textile assemblies with complex morphology used in firefighters’ multilayer protective clothing were designed in a CAD environment. The main goal of the research was to design and experimentally verify (by thermography) the models in terms of simulations when the heat transfer occurs through them in selected ambient conditions using the finite volume method. The designed models took into account the subtle differences in the geometry of selected assemblies determined by high-resolution X-ray microtomography. The designed models made it possible to calculate heat transport with a difference of about 2% to 5% in comparison to experiment that depend on the ambient conditions and the complexity of the model geometry. Moreover, the comparison of the simulation results with the experimental outcomes shows that the mapping of subtle differences in the internal structure of the assemblies in the designed models allows us to observe differences in the modeled heat transfer.

## 1. Introduction

**Figure 1:**
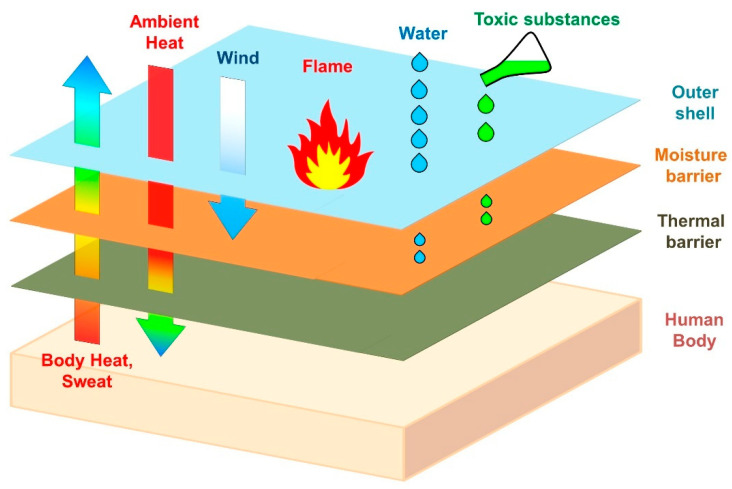
Scheme of the construction of a multilayer assembly used in firefighters’ protective clothing.

Firefighters often work in difficult environmental conditions. During rescues and other firefighting actions, as well as during the removal of the debris of accidents and natural disasters, they are exposed to many factors that adversely affect their health and life. Hot surfaces, air, and other gases can cause burns to the skin and respiratory tract, and falling structural elements of buildings, devices, and installations can cause numerous injuries in the event of impact or crushing. In addition, firefighters are exposed to electric shocks from damaged devices, installations, and electric lines, and toxic substances escaping from leaky tanks and technological installations can lead to acute poisoning. Firefighters are also exposed to the effects of both high (flames, thermal radiation) and negative temperatures (e.g., rescue operations carried out in frozen waters). In the first case, firefighters risk overheating their body and suffering from burns; in the second, they risk frostbite, bronchitis, or pneumonia. During their dangerous work, firefighters must protect themselves and others, and therefore they should be maximally prepared in terms of physical and mental fitness, unrestricted in their movement, and able to fully concentrate. One of the solutions to these conditions is to ensure thermal comfort, i.e., a correct heat balance between the firefighter’s body and the environment in which firefighters work. Ensuring thermal comfort influenced by protective clothing is essential to the protection of the body against high temperatures. Conversely, the same clothing may be an obstacle in proper heat exchange between the body and the environment and constitute a barrier against the transfer of sweat produced by the firefighter’s body during physical exertion [1]. In Figure 1, a scheme of the construction of a multilayer textile assembly used in firefighters’ protective clothing is presented.

The assembly usually consists of three basic layers made of flame-resistant textiles with different purposes: (1) outer shell, (2) moisture barrier, and (3) thermal barrier. The outer shell is the most durable element of a firefighter’s suit and protects against heat and flames as well as toxic substances and water. Moreover, it protects against the effects of mechanical impacts from the environment, such as abrasions and cuts. The moisture barrier is the middle layer of a fire suit and provides protection against the ingress of water and chemicals that partially penetrate through the outer shell. The moisture barrier also plays a crucial role in the breathability and insulation of the overall suit. It usually consists of two elements: a waterproof, vapor-permeable membrane and a substrate (e.g., a nonwoven fabric) to which the membrane is connected to. The thermal barrier is the innermost layer and is made of a heat-insulating material (nonwoven fabric or foam) and lining (woven fabric) that provides thermal insulation by creating air gaps and microclimate regions inside the suit to ensure comfort and minimize heat stress. In addition, it wicks moisture (sweat) away from the body, which improves the firefighter’s biophysical comfort. The smooth lining makes the suit easy to put on and to take off [2].

Heat transfer through protective clothing is primarily influenced by the temperature difference between the firefighter’s body and the environment in which they are working, as well as the geometric, thermal, and sorption parameters of the textile layers from which the clothing is made (layer thickness, porosity, density, specific heat, thermal conductivity, moisture sorption, vapor permeability).

The impact of the aforementioned parameters of protective clothing on the firefighter’s comfort and safety is the subject of in-depth research [3,4,5,6,7,8,9,10,11,12,13,14,15,16,17,18,19,20,21,22,23,24,25]. According to [3], the biophysical comfort properties of firefighters’ protective clothing can be improved with the incorporation of superabsorbent fibers into the inner fabric layer. Research on the critical impact of thermal liners in thermal protective performance for firefighter gear showed the positive influence of incorporating aerogel and microencapsulated phase change materials (PCMs) on thermal properties of protective clothing [5]. The use of aerogel and PCMs can also be an effective solution for improving the protective properties of various types of functional clothing and actively adapting its thermal insulation to the changing temperature conditions of the working environment [6]. Research [18] in which the influence of the thickness of the air gap on the thermal protection of protective clothing against hot steam and heat radiation showed that the air gap between clothing and skin is conducive and provides better thermal protection during exposure to heat radiation.

Currently, the model designed in the CAD environment, as well as numerical simulations, are used increasingly often as an effective tool for the analysis of physical processes affecting the utility comfort of clothing. The processing power of computers, which has grown over the years, makes it possible to design increasingly accurate models of textiles that reflect the structure of real materials with greater precision and allow for the ability to predict their key properties for a given application [26,27,28,29,30,31,32,33,34,35,36,37,38].

The research described in the current article is a continuation of research on the modeling of physical phenomena occurring in clothing [39,40,41,42,43,44,45,46]. The subjects of the research were multilayer textile assemblies used in firefighting protective clothing of similar geometry and composition. The tested assemblies were made of flame-resistant woven fabrics, nonwovens, knitted fabrics, and membranes. Using the selected CAD software, three-dimensional assembly models were designed in which heat transport was then simulated in selected environmental conditions. In order to accurately map the spatial geometry of the designed models, the geometric parameters of real textiles were determined using high-resolution X-ray microtomography (micro-CT). In addition to the precise determination of parameters such as layer thickness or the orientation and size of yarns (in woven and knitted fabrics), the micro-CT measurements allowed for the precise determination of the air content (porosity) and its distribution in textile materials, which are the key factors that determine the thermal insulation properties of the assemblies. Moreover, in the case of woven fabrics and knitted fabrics, it was possible to determine the yarn porosity in addition to total porosity, much like in nonwovens and membranes. Accurate measurements of porosity allow for the use of homogenization without having to map the complicated pattern of fibers in the yarn of woven and knitted fabrics and in the total volume of the nonwovens. The results of the heat transport simulation through assembled models were verified by an experiment carried out in identical environmental conditions with real textiles using a hotplate and thermal imaging camera.

## 2. Materials and Methods

### 2.1. Materials

**Figure 2:**
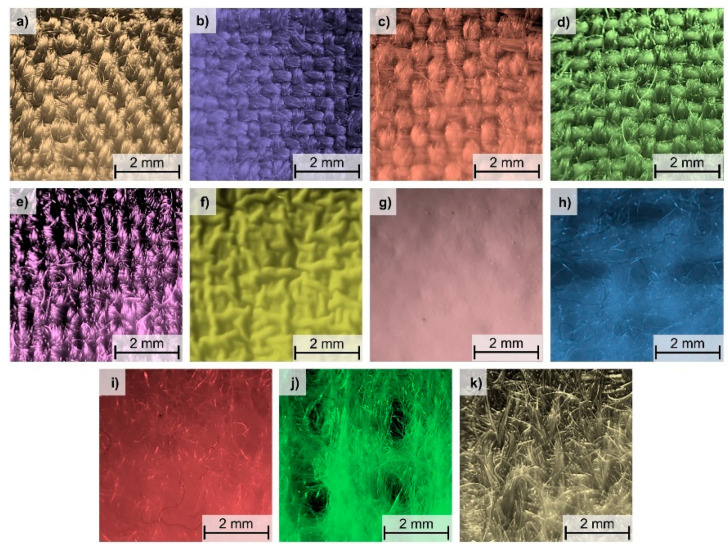
Optical microscopy images: (**a**) woven fabric 1; (**b**) woven fabric 2; (**c**) woven fabric 3; (**d**) woven fabric 4; (**e**) woven fabric 5; (**f**) membrane 1; (**g**) membrane 2; (**h**) nonwoven fabric 1; (**i**) nonwoven fabric 2; (**j**) nonwoven fabric 3; (**k**) nonwoven fabric 4.

The subjects of the investigations were four five-layer textile assemblies (*A–D*) of similar composition and different geometry. Physical parameters of the assemblies were presented in Table 1, whereas optical microscopy images of all textiles constituting the assemblies, obtained using an optical microscope (PZO, Warsaw, Poland) equipped with a digital optical camera (DLT-Cam PRO, Delta Optical, Warsaw, Poland) and software (DLT CamViewer), were presented in Figure 2. The assembly layers consisted of the following eleven textiles: five woven fabrics (made with aramid), two membranes (made with polyurethane), and four nonwoven fabrics (made with aramid). In all tested assemblies, the outer shell was made of woven fabric, the moisture barrier was made of membranes inseparably connected with nonwoven fabric, and the thermal barrier was built of inseparably connected nonwoven and woven fabrics. The applied woven fabrics differed in thickness, weave, weft density, warp density, porosity, and yarn porosity.

The membranes and nonwoven fabrics differed in thickness and porosity. Two nonwoven fabrics (nonwoven fabric 1 and nonwoven fabric 3) were characterized by a periodic arrangement of openings open on both sides of the layer, while in nonwoven fabric 3 there was also a periodic arrangement of shallower openings on one side of the layer. Both types of openings (visible on Figure 2h,j) were filled with air, which ensured greater thermal insulation for the nonwoven fabrics. Inside nonwoven fabric 4, there was a reinforcing layer in the form of a thin knitted fabric of single jersey weave (visible on Figure 2k) made of the same raw material as nonwoven fabric 4.

Based on the characteristics of the assemblies presented in Table 1, it can be observed that assemblies *A* and *C* as well as *B* and *D* are very similar; assemblies *A* and *C* differ only in the woven fabric of the outer shell, while assemblies *B* and *D* differ only in the structure of the membrane. As a result, assemblies A and C and assemblies *B* and *D* have a comparable thickness. This particular selection of tested textiles was made to investigate the influence of slight differences in the internal structure (determined using high-resolution micro-CT) of the multilayer assemblies on their thermal insulation properties. Moreover, one of the goals of the simulation was to investigate the influence of subtle changes in the geometry of three-dimensional assembly models on the calculated heat transfer under selected thermal conditions.

### 2.2. Methods

#### 2.2.1. X-ray Microtomography (Micro-CT)

**Figure 3:**
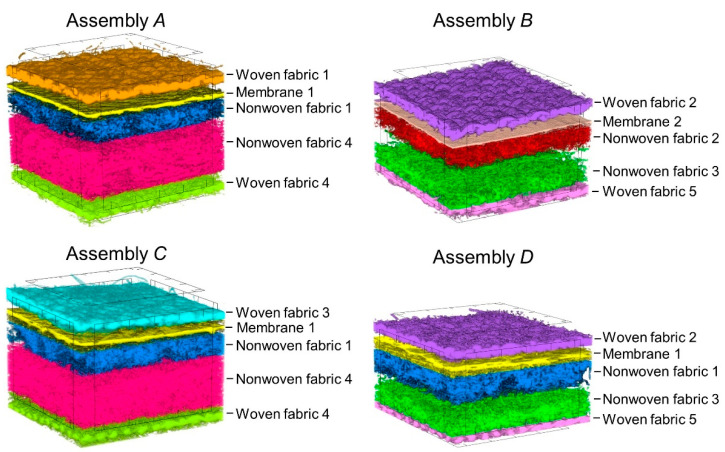
3D micro-CT reconstructions of tested assemblies (dimensions: 4 mm × 4 mm).

To accurately reflect the spatial geometry of the tested textiles in 3D models, high-resolution X-ray microtomography (SkyScan 1272; Bruker, Kontich, Belgium) was applied. Structural parameters of tested assemblies (presented in Table 1) were obtained using the following scanning conditions: X-ray source voltage 50 kV, X-ray source current 200 µA, and pixel size 5 µm. A 180° rotation was applied with a rotation step of 0.2° without using a filter. Calculations of geometrical parameters of tested textiles were performed using CTAn 1.17.7.2+ software (made by Bruker, Kontich, Belgium). 3D reconstructions of the assemblies (presented in Figure 3) were prepared using NRecon 1.7.4.2 and CTvox 3.3.0 r1403 software (made by Bruker, Kontich, Belgium).

X-ray microtomography enables the analysis of the internal structure (in a micro scale) of the tested material based on the contrast of different areas of the three-dimensional reconstruction. This occurs as a result of the different values of X-ray absorption by the elements of the scanned object due to the difference in their density. Namely, the X-ray absorption of aramid fiber differs from the X-ray absorption of air filling the space between the fibers. Aramid, due to its greater density than air density, absorbed X rays more than air, making it possible to calculate the air content in a textile material (porosity).

#### 2.2.2. Thermal Imaging

**Figure 4:**
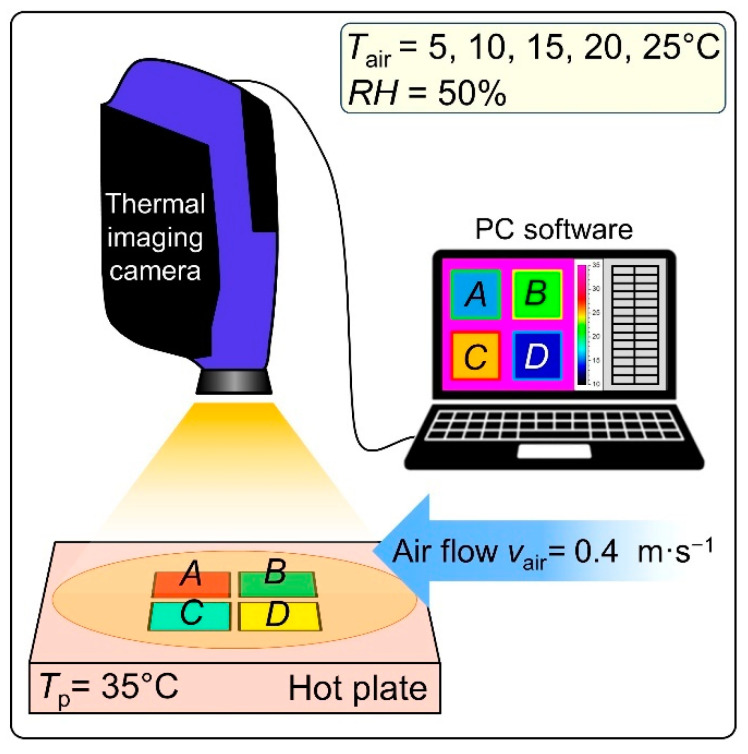
Scheme and initial conditions of thermal imaging.

Thermal imaging measurements were performed in an airconditioned room (Great Climatic Chamber made by Weiss Technik, Oldenburg, Germany) that allowed for the precise regulation of air temperature (*T*_air_) and relative air humidity (*RH*). The scheme of the measurement system is shown in Figure 4; it consists of a hot plate (e-G51HP07C Guardian 5000 model made by OHAUS Europe GmbH, Nänikon, Switzerland) and a thermal imaging camera (FLIR SC 5000 model made in Wilsonville, OR, USA).

The applied hot plate allows for the regulation and control of the temperature, with an accuracy of ±1 °C, on the entire surface of the flat plate. During the measurements, the four tested assemblies (*A*, *B*, *C*, *D*) were placed (without any additional mechanical load) on the hot plate with the woven fabric (outer shell) facing upwards, while the woven fabric (thermal barrier) was directly adjacent to the hot plate. The hot plate temperature (*T*_p_) was always higher than the air temperature in the airconditioned room, constant over time, and similar to the temperature of human skin at 35 °C. As a result of the temperature difference between the hot plate and the surroundings, heat transfer (mainly by conduction) was generated from the hot plate to the cooler environment through the tested assemblies. To test the thermal insulation properties of the textiles, the average temperature of the top surface (*T*_top_) of every assembly was measured using a thermal imaging camera (controlled by Altair—Thermal Image Analysis Software) placed above the hot plate. The measurements were performed for five selected constant values of air temperature: *T*_air_ = 5 °C, *T*_air_ = 10 °C, *T*_air_ = 15 °C, *T*_air_ = 20 °C, *T*_air_ = 25 °C; constant value of relative air humidity: *RH* = 50% and constant value of air pressure: *p*_air_ = 1013.25 hPa. An additional factor influencing the results of the experiment was the horizontal air flow with a constant velocity of 0.4 m s^–1^, which caused the more effective cooling of the upper layer of the assemblies as a result of forced convection. The tested assembly samples were in the form of a cuboid with horizontal dimensions of 20 mm × 20 mm; however, to eliminate the negative influence of boundary conditions, the analyzed surface area was reduced to a square of 10 mm × 10 mm. For all five selected ambient conditions, the measurements were performed after reaching a steady state (temperature of top assembly layer was constant in time).

#### 2.2.3. Model Designing

**Figure 5:**
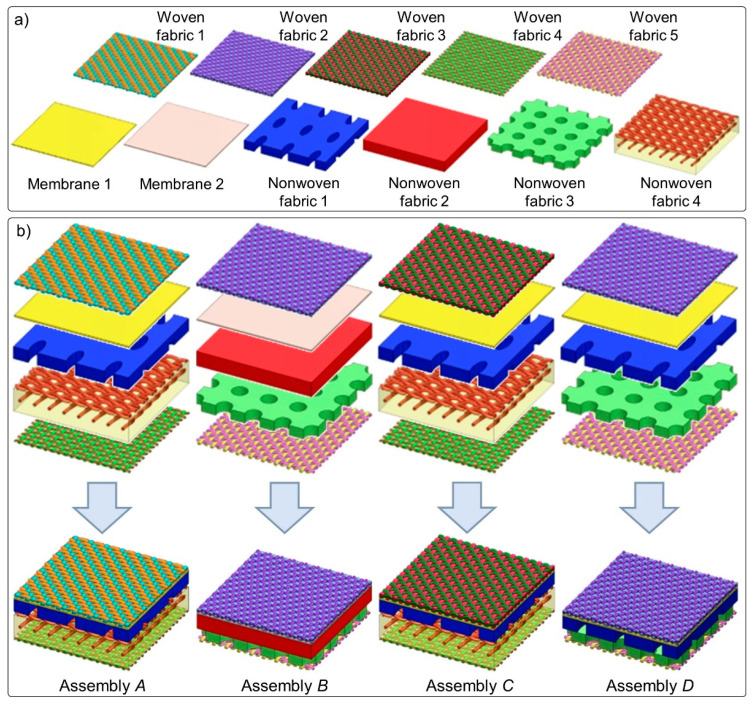
(**a**) 3D models of all eleven textiles included in the four tested assemblies; (**b**) 3D models of four tested assemblies (dimensions: 10 mm × 10 mm).

On the basis of geometric parameters determined using X-ray micro-computed tomography and optical microscopy, three-dimensional models of the all eleven textiles included in the four tested assemblies (Figure 5a) were designed using SolidWorks 2014 CAD software (Dassault Systèmes, Waltham, MA, USA) [45]. The method of designing textiles models with the use of selected software has been described in detail in earlier studies by the authors [39,40,41,42]. The following six geometric parameters were taken into account in the models of all five woven fabrics: (1) thickness, (2) weave, (3) warp density, (4) weft density, (5) yarn thickness, and (6) elliptic shape of cross-section of warp and weft yarns. In the case of knitted fabric inside nonwoven fabric 4, the following five geometrical parameters were mapped: (1) thickness, (2) weave, (3) loop shape, (4) distance between loops, and (5) elliptic shape of cross-section of yarn. The yarns in all five of the woven fabrics models and knitted fabric inside nonwoven fabric 4 model were designed as monofilaments (fibers in the yarn and the spaces between them have not been mapped). Two membranes and four nonwoven fabrics were mapped as cuboidal objects with measured thickness. For the model of nonwoven fabric 1, a periodic arrangement of openings open on both sides of the nonwoven fabric was mapped; for the model of nonwoven fabric 3, a periodic arrangement of openings was also mapped, but the openings are shallower and do not extend throughout the whole nonwoven fabric layer.

The designed textile models were based on the following additional simplifications. It was assumed that, in the case of woven fabrics and knitted fabric, the following parameters are constant in the entire volume of the textile: layer thickness, weave, warp density, weft density, yarn thickness, and yarn porosity. Additionally, it was assumed that, in the case of knitted fabric, layer thickness, weave, loop shape, distance between loops, and the elliptic cross-section of yarn, yarn thickness, and yarn porosity are constant in the entire volume of the textile. In real textiles, the yarn thickness and the shape of the cross-section of the yarn is not constant because, as a result of the friction of adjacent yarns, their deformation occurs. Moreover, it was assumed that layer thickness and porosity in nonwoven fabrics and membranes are constant in the entire volume of the textile. Micro-CT measurements showed that, in real nonwovens and membranes, there are areas with different layer thickness and density of matter. For this reason, in the yarn of woven fabrics and the yarn of knitted fabrics, air homogenization with the raw material consisting of fibers was applied with mutual proportions as a result of the calculated average porosity of these yarns. Similarly, in nonwoven fabrics, air homogenization with the raw material consisting of the nonwovens was used with mutual proportions resulting from the calculated average porosity of these textiles. From the designed five models of woven fabrics, two models of membranes and four models of nonwoven fabrics in addition to four models of multilayer assemblies were built (Figure 5b). Another simplification was the assumption that all adjacent layers making up the assembly adhere to each other without causing air gaps between the layers. In the assembly models, single threads connecting individual layers were not mapped due to their low weight and thus negligible impact on the thermal insulation properties of the multilayer assemblies. 

#### 2.2.4. Heat Transfer Simulations

##### Physical Basis

Woven fabrics, nonwoven fabrics, and knitted fabrics present in the tested assemblies are textiles composed of interconnected systems of fibers between which there is space filled with air. Therefore, the heat transport in these materials propagates both through the fibers (solid) and air (gas). The applied software (Solidworks Flow Simulation 2014) allows us to calculate the conduction within fluid and solids applying Computational Fluid Dynamics (CFD). The software calculates heat transfer in fluids using Navier–Stokes equations, which are formulations using mass, momentum, and energy conservation laws for fluid flows. The equations are supplemented by fluid state equations defining the nature of the fluid and by empirical relationships of fluid density, viscosity, and thermal conductivity on temperature. Whereas heat conduction in solids is described by the following Equation [48]:

$$\frac{\partial \left(\rho e\right)}{\partial t}=\frac{\partial }{\partial x_{i}}\left(\lambda_{i}\frac{\partial T}{\partial x_{i}}\right)+Q_{H}$$

where *ρ* is solid density [kg·m^−3^] and *e* is the specific internal energy, *e = c·T* [J], *c* is specific heat [J∙kg^−1^∙K^−1^], *T* is temperature [K], *Q*_H_ is specific heat release (or absorption) rate per unit volume [W·m^−3^], and *λ*_i_ are the eigenvalues of the thermal conductivity tensor [W·m^−1^·K^−1^]. A more detailed description of the physical basis of which the simulations were performed was shown in an earlier work on the modeling of the thermal performance of firefighters’ protective clothing that is exposed to radiant heat [43] and flames [44].

##### Conditions of Heat Transfer Simulations

**Figure 6:**
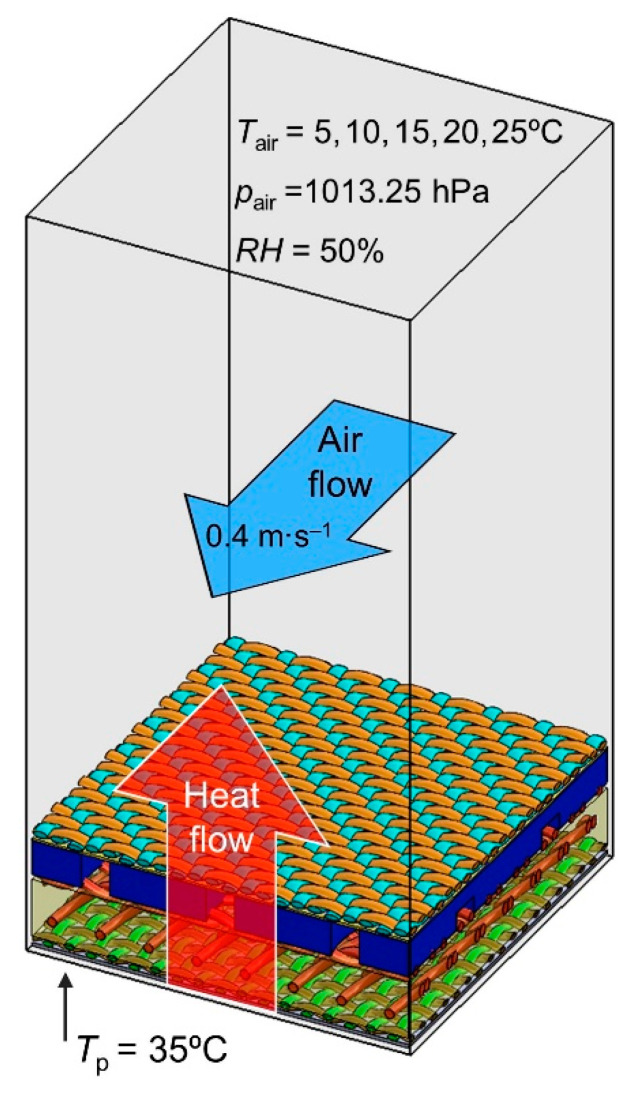
Initial conditions of simulations of heat transfer through assembly model.

The main goal of the heat transfer simulations was to calculate the average temperature of the top surface (*T*_top_) of each of the four assembly models in identical conditions under which the experiment was carried out on real assemblies using thermal imaging (constant *T*_p_, five different values of *T*_air_, constant *RH*, and constant airflow velocity). Therefore, each of the four assembly models (reduced to an area of 10 mm × 10 mm) was placed on a plate model (cuboid of identical size) with a constant temperature of 35 °C. The assembly model and plate model were placed on the bottom of the computational domain (10 mm × 10 mm × 20 mm cuboid) and filled with air (Figure 6). To eliminate the negative influence of boundary conditions on the accuracy of calculation results, periodic boundary conditions were applied to simulate the continuity of the model assembly outside the computational domain. Before starting the calculations, the interior of the computational domain was filled with a mesh of finite volume elements, i.e., three types of cells: solid (containing the assembly model and the plate model), gas (containing air), and partial (containing the assembly model or the hot plate model and air). The number and size of cells was different for each assembly model as a result of its height and the different geometry of the textile models making up the assembly model (Table 2).

Based on the results of computed microtomography, the values of porosity of all textiles (woven fabrics, membranes, woven fabrics, knitted fabric) forming the four tested assemblies were determined. In addition, for all five woven fabrics and the knitted fabric inside nonwoven fabric 4, the porosity of the yarn was also calculated. Based on these calculations, homogenization of the raw material [40,41] from which these textiles were made (presented in Table 3) with air was applied to all models of nonwovens, both membrane models, yarns in the models of woven fabrics, and yarns in the knitted fabric model, with mutual proportions resulting from the calculated porosity of these materials. Through this method, the calculated air content inside the actual textiles was taken into account without having to map the complex pattern of fibers in the woven yarns, knitted yarns, and nonwovens. 

The simulation results, like the measurement results in the experiment, were obtained under the steady state conditions.

## 3. Results

**Figure 7:**
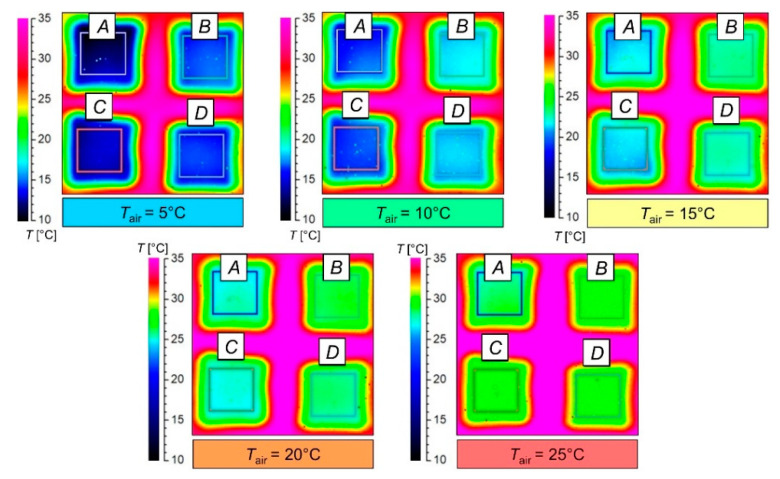
Thermograms of tested assemblies obtained in five different ambient conditions (analyzed surface area: 10 mm × 10 mm—indicated by square).

Figure 7 shows the thermograms obtained in the experiment carried out in the five selected ambient temperatures (*T*_air_) on the basis of which the average temperature of the top surface (*T*_top_) of the four tested assemblies were calculated. The sample area (10 mm × 10 mm square) was marked on each thermogram, on the basis of which the average *T*_top_ value was calculated. A separate temperature scale was attached to each thermogram because the software controlling the thermal imaging camera updated the color palette in real time during the experiment for the best fit. 

**Figure 8:**
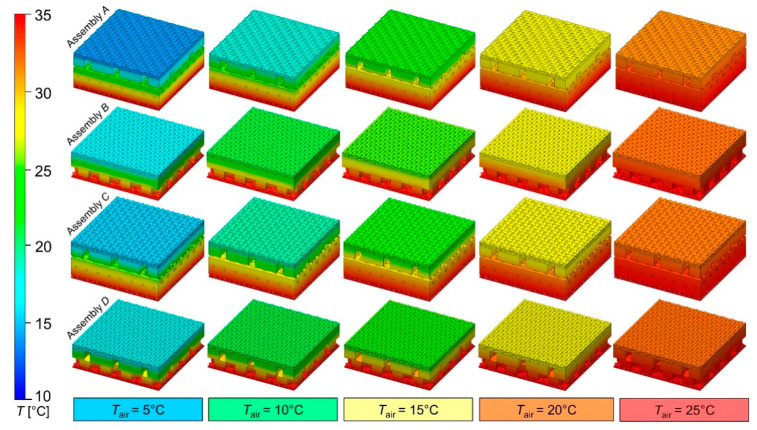
Simulated temperature distributions on 3D models of tested assemblies (surface area: 10 mm × 10 mm).

Figure 8 shows the temperature distributions on 3D models of the tested assemblies. The distributions were obtained using heat transfer simulations carried out in the five selected ambient temperatures (*T*_air_). 

**Figure 9:**
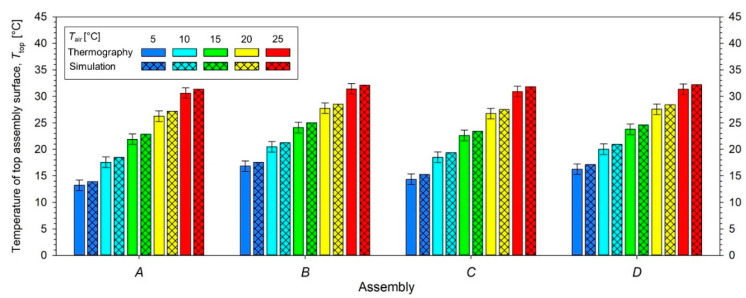
Experimental and simulated temperatures of top assembly surface *T*_top_ in selected ambient conditions (for the experimental results, the range of measurement error of *T*_top_ (±1 °C), which is a result of the specification of the thermal imaging camera, has been marked on thermography bars).

On the outer vertical walls of all 4 assembly models, a vertical linear temperature drop (from hot plate to the surroundings) is visible, which confirms the correct application of the boundary conditions applied to the computational domain. Experimental and modeled *T*_top_ values were presented in Figure 9 and Table 4. The experimental and modeled *T*_top_ values for each tested assembly correlate with each other and increase when increasing ambient temperature *T*_air_. For each tested assembly, the modeled *T*_top_ values are always slightly higher than the corresponding temperatures obtained in the experiment, but the difference is always within the range of the measurement error (±1 °C), which is a result of the specification of the thermal imaging camera (marked in form error bar in Figure 8). The computational error range resulting from applying the finite volume mesh density (the number of cells inside the computational domain) was ±0.01 °C and, due to its small value, it was not presented on Figure 9 as an error bar; instead, it is presented in Table 4. Additionally, Table 4 presents the experimental and modeled values of the temperature drop in the assembly *D*_T_ = *T*_p_ − *T*_top_, i.e., the difference between the constant temperature of the hot plate, *T*_p_ = 35 °C, and the temperature of top assembly surface, *T*_top_ (variable depending on environmental conditions).

**Figure 10:**
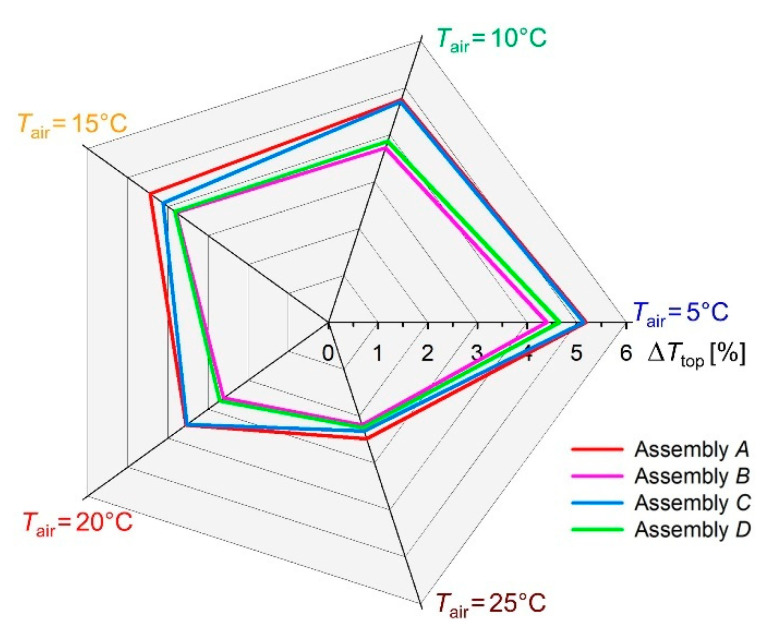
Percentage difference between experimental and simulated temperature of top assembly surface Δ*T*_top_ obtained for 5 environmental conditions.

However, the difference between the experimental and simulated value of *T*_top_ increases by increasing the difference between the constant hot plate temperature (*T*_p_ = 35 °C) and the ambient temperature (*T*_air_). To better present this effect, a radar plot (Figure 10) was prepared, which shows the difference, Δ*T*_top_ [%], between the *T*_top_ temperatures measured in the experiment and the simulation at the selected five ambient temperatures *T*_air_.

As can be seen from the radar chart, the greatest differences Δ*T*_top_ range from 4.40% (Assembly *B*) to 5.18% (Assembly *A*) and were observed for an ambient temperature *T*_air_ = 5 °C; the smallest differences Δ*T*_top_ range from 2.19% (Assembly *B*) to 2.48% (Assembly *A*) were obtained for an ambient temperature *T*_air_ = 25 °C. In general, it can be concluded that the assembly models are less thermally insulating than their real counterparts. The uneven distribution of temperature on the top surface of the tested assemblies, visible on the thermograms (Figure 7), may indicate that the distribution of matter in the individual layers is non-uniform and that these layers do not adhere to each other over the entire tested surface (which causes local air gaps). These two factors contribute to the unequal heat transfer through the assemblies.

## 4. Discussion

**Figure 11:**
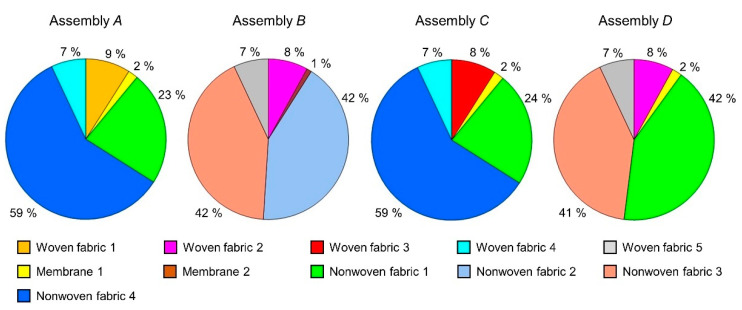
Percentage heat loss in layers of four tested assemblies.

Apart from the temperature of top assembly surface *T*_top_, the results of the heat transfer simulation allowed for the analysis of other parameters of the modeled phenomenon. On the basis of the temperature distribution over the thickness of each assembly (Figure 9), the heat losses in each layer of the assembly were calculated and presented in the form of pie charts in Figure 11. 

**Figure 12:**
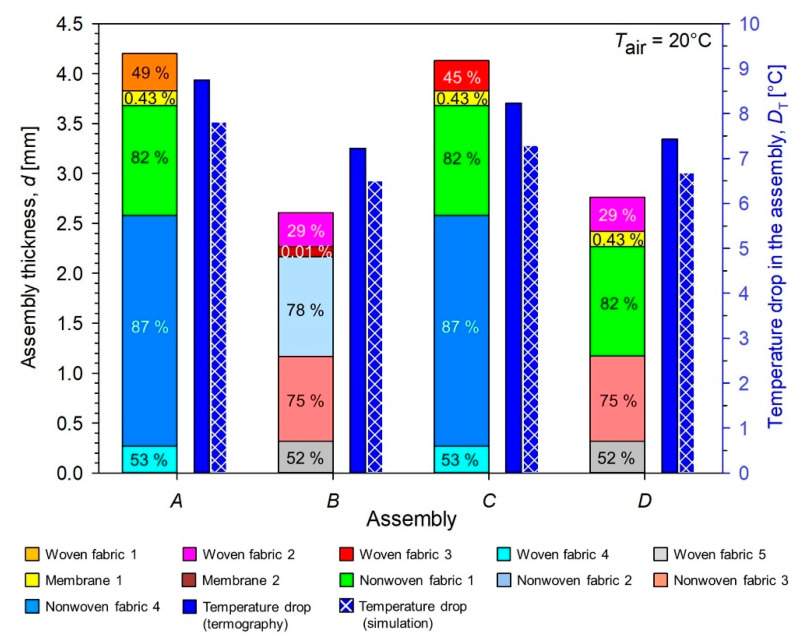
Influence thickness *d* and porosity *p* (presented inside wide bars) of assembly layers on experimental (blue plain bar) and simulated (blue checked bar) drop temperature *D*_T_ in the assembly for air temperature *T*_air_ = 20 °C.

Similar calculations were not possible on the basis of experimental data due to the insufficient spatial resolution of the image obtained with the use of a thermal imaging camera; however, slight differences of *T*_top_ occurred between the simulations and the experiment and it can be assumed that the simulated heat losses are close to the corresponding experimental values. The pie charts show that, in the case of all tested assemblies, nonwovens constituted the greatest thermal protection. In the case of assemblies *A* and *C*, the nonwovens included in the moisture barrier (nonwoven fabric 1) and thermal barrier (nonwoven fabric 4) retained 82% and 83% of the heat passing through all layers of the assembly, respectively. For assemblies *B* and *D*, the nonwovens included in the moisture barrier (nonwoven fabric 2 in Assembly *B*, nonwoven fabric 1 in Assembly *D*) and thermal barrier (nonwoven fabric 3) retained 84% and 83% of the heat passing through all layers of the assembly, respectively. The second obstacle in terms of the amount of heat retained were woven fabrics. As can be seen in pie charts for assemblies *A* and *C*, the woven fabrics included in the outer shell (woven fabric 1 in Assembly *A*, woven fabric 3 in Assembly *B*) and thermal barrier (woven fabric 4) retained 16% and 15% of the heat passing through all layers of the assembly, respectively. In case of assemblies *B* and *D*, the woven fabrics included in the outer shell (woven fabric 2) and thermal barrier (woven fabric 5) retained 15% of the heat passing through all layers of the assembly. The least thermally insulating layer in all tested assemblies were membrane 1 and membrane 2, which were part of the moisture barrier. Membrane 1, present in assemblies *A, C, D*, retained 2% of heat, while membrane 2, present in Assembly *B*, retained 1% of heat. In Figure 12, the influence thickness *d* and porosity *p* (presented inside wide bars) of assembly layers on experimental (blue plain bar) and simulated (blue checked bar) drop temperature *D*_T_ in the assembly (for air temperature *T*_air_ = 20 °C) was presented. 

In the case of assemblies *A* and *C*, a slight difference (1%) in heat loss in nonwoven fabric 1 was likely due to the presence of different fabrics in the outer shell. The thicker (*d* = 0.37 mm) and more porous (*p* = 49%) woven fabric 1 present in Assembly *A* had a greater resistance to heat flowing through the assembly than the thinner (*d* = 0.33 mm) and less porous (*p* = 45%) woven fabric 3. In the case of assemblies *B* and *D*, the little difference (1%) in heat loss in nonwoven fabric 3 was probably cause by the use of different membranes in the moisture barrier. The thicker (*d* = 0.15 mm) and more porous (*p* = 0.43%) membrane 1 present in Assembly *B* had a greater resistance to heat flowing through the assembly than the thinner (*d* = 0.10 mm) and less porous (*p* = 0.01%) membrane 2.

## 5. Conclusions

The main purpose of this study was to model the heat transport through the four textile assemblies used in multilayer firefighting clothing of similar geometry and raw material composition. First, in an experiment involving a hot plate and a thermal imaging camera, measurements of heat transport through the assemblies were carried out under five selected environmental conditions. Next, the geometric parameters of the assemblies (made of woven fabrics, nonwoven fabrics, membranes, and knitted fabrics) were precisely determined on the basis of high-resolution X-ray microtomography and, on their basis, three-dimensional models were designed using CAD software. Based on the microscale porosity measurements of the tested textiles using high-resolution X-ray tomography, homogenization was applied without the need to reproduce the complex arrangement of fibers in woven fabrics, knitted fabrics and nonwoven fabrics. Then, simulations of heat transfer through textile models were conducted using the finite volume method in identical environmental conditions in which the experiment was carried out. A comparison of the results obtained in the simulations and the experiment verifying them can be stated:

- Mapping the subtle differences in the internal structure (spatial geometry, porosity) of the assemblies in the designed models measured using micro-CT reveals observable differences in the modeled heat transfer.
- Despite applied simplifications in geometry and the use of homogenization, the designed assembly models make it possible to predict heat transfer through real assemblies with a difference of about 2% to 5% in comparison to the experiment depending on the environmental conditions (the difference increases with the temperature difference inducing heat transfer) and the complexity of the model geometry. Generally speaking, simplifications in mapping the complex geometry of the slightly different assemblies did not affect the sensitivity for detecting differences in the modeled heat transfer that resulted from minimal differences in the morphology of the tested textiles.
- The applied design and simulation method is a useful tool for the accurate modeling and prediction of heat transfer through multilayer textiles with complex geometry before the production of clothing, which results in savings in the raw materials, energy, labor costs, and time needed to produce them.

## Figures and Tables

**Table 1:** Characteristics of four tested assemblies.

Assembly	Layer Type	Textile	Weave	Weft/Warp Density [cm^–1^]		Textile Composition	Textile/Assembly Thickness ^a)^ [mm]		Surface Mass ^b)^ [g·m^–2^]	Textile/Assembly Porosity ^a)^ [%]		Yarn Porosity ^a)^ [%]
*A*	Outer shell	Woven fabric 1	twill	22	22	aramid	0.37	4.20	208.27	49.38	77.51	26.88
	Moisture barrier	Membrane 1	none	none		polyurethane	0.15		182.64	0.43		none
		Nonwoven fabric 1	none	none		aramid	1.10			82.20		none
	Thermal barrier	Nonwoven fabric 4	none	none		aramid	2.31		278.74	87.60		47.82 *
		Woven fabric 4	plain	18	18	aramid	0.27			53.52		24.66
*B*	Outer shell	Woven fabric 2	twill	29	29	aramid	0.34	2.61	202.44	28.70	66.00	12.44
	Moisture barrier	Membrane 2	none	none		polyurethane	0.10		149.02	0.01		none
		Nonwoven fabric 2	none	none		aramid	1.00			78.27		none
	Thermal barrier	Nonwoven fabric 3	none	none		aramid	0.85		198.82	74.65		none
		Woven fabric 5	plain	18	18	aramid	0.32			52.26		25.52
*C*	Outer shell	Woven fabric 3	plain	18	18	aramid	0.33	4.16	194.25	45.39	77.47	19.67
	Moisture barrier	Membrane 1	none	none		polyurethane	0.15		182.64	0.43		none
		Nonwoven fabric 1	none	none		aramid	1.10			82.20		none
	Thermal barrier	Nonwoven fabric 4	none	none		aramid	2.31		278.74	87.60		47.82 *
		Woven fabric 4	plain	18	18	aramid	0.27			53.52		24.66
*D*	Outer shell	Woven fabric 2	twill	29	29	aramid	0.34	2.76	202.44	28.70	67.22	12.44
	Moisture barrier	Membrane 1	none	none		polyurethane	0.15		182.64	0.43		none
		Nonwoven fabric 1	none	none		aramid	1.10			82.20		none
	Thermal barrier	Nonwoven fabric 3	none	none		aramid	0.85		198.82	80.61		none
		Woven fabric 5	plain	18	18	aramid	0.32			52.26		25.52

^a)^ determined using X-ray micro-computed tomography ^b)^ determined according to PN EN 12127:2000 [47] * yarn porosity of knitted fabric inside nonwoven fabric 4.

**Table 2:** Number of cells in tested assembly models.

Assembly	Solid Cells	Gas Cells	Partial Cells
*A*	744,868	455,061	363,352
*B*	111,964	71,474	61,362
*C*	779,686	425,528	359,839
*D*	114,583	87,274	72,577

**Table 3:** Physical features of raw materials applied in heat transfer simulations through tested assembly models.

Physical Parameter	Aramid	PU	Air
density [kg·m^−3^]	1360	1260	1.2
specific heat [J·kg^−1^·K^−1^]	1390	1120	1005
thermal conductivity [W·m^−1^·K^−1^]	0.18	0.23	0.03

**Table 4:** Experimental and simulated temperatures of top assembly surface *T*_top_ and temperature drops *D*_T_.

Assembly	*T*_air_ [°C]	Temperature of Top Surface, *T*_top_ [°C]		Temperature Drop, *D*_T_ [°C]	
		Thermography	Simulation	Thermography	Simulation
*A*	5	13.21 ± 1	13.89 ± 0.01	21.79 ± 1	21.11 ± 0.01
	10	17.56 ± 1	18.39 ± 0.01	17.44 ± 1	16.61 ± 0.01
	15	21.91 ± 1	22.88 ± 0.01	13.09 ± 1	12.12 ± 0.01
	20	26.26 ± 1	27.19 ± 0.01	8.74 ± 1	7.81 ± 0.01
	25	30.61 ± 1	31.37 ± 0.01	4.39 ± 1	3.63 ± 0.01
*B*	5	16.83 ± 1	17.57 ± 0.01	18.17 ± 1	17.43 ± 0.01
	10	20.48 ± 1	21.24 ± 0.01	14.52 ± 1	13.76 ± 0.01
	15	24.13 ± 1	25.04 ± 0.01	10.87 ± 1	9.96 ± 0.01
	20	27.77 ± 1	28.50 ± 0.01	7.23 ± 1	6.50 ± 0.01
	25	31.42 ± 1	32.11 ± 0.01	3.58 ± 1	2.89 ± 0.01
*C*	5	14.35 ± 1	15.09 ± 0.01	20.65 ± 1	19.91 ± 0.01
	10	18.49 ± 1	19.36 ± 0.01	16.51 ± 1	15.64 ± 0.01
	15	22.63 ± 1	23.56 ± 0.01	12.37 ± 1	11.44 ± 0.01
	20	26.77 ± 1	27.71 ± 0.01	8.23 ± 1	7.29 ± 0.01
	25	30.90 ± 1	31.62 ± 0.01	4.10 ± 1	3.38 ± 0.01
*D*	5	16.25 ± 1	17.01 ± 0.01	18.75 ± 1	17.99 ± 0.01
	10	20.03 ± 1	20.80 ± 0.01	14.97 ± 1	14.20 ± 0.01
	15	23.80 ± 1	24.71 ± 0.01	11.20 ± 1	10.29 ± 0.01
	20	27.57 ± 1	28.32 ± 0.01	7.43 ± 1	6.68 ± 0.01
	25	31.35 ± 1	32.05 ± 0.01	3.65 ± 1	2.95 ± 0.01
